# Linearly Sensitive and Flexible Pressure Sensor Based on Porous Carbon Nanotube/Polydimethylsiloxane Composite Structure

**DOI:** 10.3390/polym12071499

**Published:** 2020-07-05

**Authors:** Young Jung, Kyung Kuk Jung, Dong Hwan Kim, Dong Hwa Kwak, Jong Soo Ko

**Affiliations:** 1 Graduate School of Mechanical Engineering, Pusan National University, Busandaehak-ro 63beon-gil, Geumjeong-gu, Busan 46241, Korea; young89@mems.ac.kr (Y.J.); akiocom55@pusan.ac.kr (D.H.K.); kwak3856@pusan.ac.kr (D.H.K.); 2 Precision Mechanical Process and Control R&D group, Korea Institute of Industrial Technology, 42-7, Baegyang-daero 804beon-gil, Sasang-gu, Busan 46938, Korea; 3 Quality & Standards Department, Korea Marine Equipment Research Institute, 435, Haeyang-ro, Yeongdo-gu, Busan 49111, Korea; kkjung@komeri.re.kr

**Keywords:** flexible pressure sensors, carbon nanotubes, linear sensitivity, composite, porous structure

## Abstract

We developed a simple, low-cost process to fabricate a flexible pressure sensor with linear sensitivity by using a porous carbon nanotube (CNT)/polydimethylsiloxane (PDMS) composite structure (CPCS). The working principle of this pressure sensor is based on the change in electrical resistance caused by the contact/non-contact of the CNT tip on the surface of the pores under pressure. The mechanical and electrical properties of the CPCSs could be quantitatively controlled by adjusting the concentration of CNTs. The fabricated flexible pressure sensor showed linear sensitivity and excellent performance with regard to repeatability, hysteresis, and reliability. Furthermore, we showed that the sensor could be applied for human motion detection, even when attached to curved surfaces.

## 1. Introduction

The development of flexible pressure sensors is gaining attention because of the requirements of the next generation of wearable systems. Flexible sensors that can detect subtle pressure induced by physical signals have found applications in various research fields such as wearable electronic devices [1,2,3], human–machine interfaces [4], and healthcare monitoring [5,6,7,8]. To use flexible sensors for wearable systems, they should be flexible and elastic so that they can be used on curved surfaces. To fulfill these requirements, polymer-based flexible sensors have been investigated. Previous studies have reported polymer-based flexible sensors utilizing piezoresistive [9,10,11,12,13], capacitive [14,15,16,17], piezoelectric [18,19], and triboelectric sensing technologies [20,21]. Among these, piezoresistive devices, which transduce pressure into a resistance signal, have been widely used because of their cost-effective fabrication, simple working principle, and easy signal processing.

Recently, conductive composites coupled with flexible substrates have been proposed to fabricate piezoresistive pressure sensors. Because the conductive fillers in soft polymer matrices lead to a change in resistance as a function of the applied pressure, the performance of such sensors is highly dependent on the conductive materials. Currently, there is a growing need to improve the properties of the sensors. Therefore, the use of nanomaterials such as carbon nanotubes (CNTs), graphene, nanofibers, and zinc oxide nanowires (ZnO NWs), as well as metallic particles that have excellent mechanical and electrical properties should be investigated for the fabrication of piezoresistive pressure sensors. Janczak et al. proposed a piezoresistive-type pressure sensor made of screen-printed polymer composites containing graphene nanoplatelets and CNTs [22]. They measured the sensing performance and analyzed the relationship between resistance and pressure according to the concentration of nanomaterials in the composites. In addition, Zhao et al. proposed a resistive pressure sensor with a hybrid composite [23]. The composite-based sensor showed favorable sensitivity with a controlled percolation threshold. However, conductive composites with high elastic modulus for the reinforcement of nanomaterials have limitations in achieving acceptable resolution over a wide pressure range due to the saturation of resistance change. Moreover, the viscoelastic behavior and barreling effect of conductive composites can cause a relatively long response time and high hysteresis.

The three-dimensional interconnected porous structure exhibits ideal performance over a wide pressure detection range with adequate sensitivity. Porous structures coated with nanomaterials have been gaining attention for their high compressibility, anti-barreling effect, low-cost fabrication method, and non-viscoelastic properties, which significantly increase their potential for use as pressure sensors with improved electromechanical properties [24,25,26,27,28]. For example, Yao et al. suggested pressure-sensitive graphene-coated polyurethane (PU) sponges with a fractured microstructure design fabricated using hydrothermal and strain deformation treatment [27]. Graphene-wrapped PU sponges were able to detect pressures as low as 9 Pa and up to 2 kPa. Song et al. also proposed highly compressible CNT-coated PDMS sponges via a sugar templating process and drop casting of CNT ink [28]. They demonstrated that these sensors were able to detect not only a subtle pressure of 26 Pa but also a high pressure of 150 kPa. Likewise, many researchers have demonstrated that porous structures can be used as flexible pressure sensors with high sensitivity and a wide detection range. However, the fabrication process based on dip coating or drop casting is expensive and requires solvents (such as ethanol and isopropyl alcohol); uniform coating of a large area is also difficult. Furthermore, the sensitivity of the porous-structure-based sensor is non-linear because of the structural limitation (i.e., change in elastic modulus due to closed pores) of the porous structure. Therefore, an additional calibration system is needed to employ such sensors in the industrial fields.

Herein, we propose a facile yet effective method to improve the sensor performance, in terms of sensitivity and linearity, of flexible pressure sensors based on a porous carbon nanotube (CNT)—polydimethylsiloxane (PDMS) composite structure (CPCS). The composite structure can be easily fabricated in large areas and has a very stable structure because of the conductive CNTs embedded in the PDMS frame. Moreover, the concentration of internal CNTs can be quantitatively controlled so CPCSs with diverse mechanical properties and high conductivity values can be fabricated. Based on the mechanical and electrical properties, we successfully achieved outstanding sensing performance with regard to linear sensitivity, repeatability, hysteresis, and reliability. In addition, the flexible pressure sensors demonstrated the capability to carry out human motion detection, such as finger and wrist bending. 

## 2. Materials and Methods

### 2.1. Preparation of Porous CNT/PDMS Composite Structure

**Figure 1:**
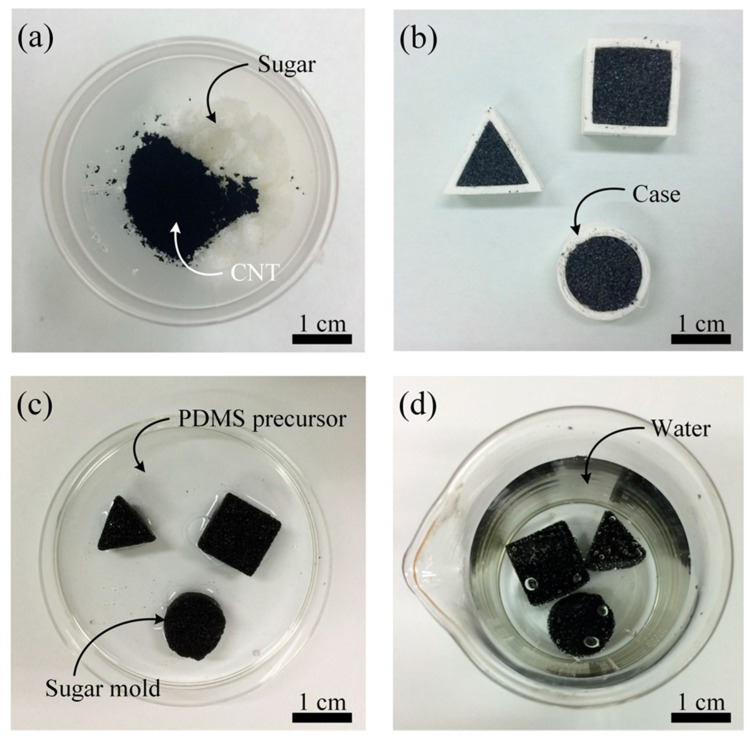
Fabrication process of carbon nanotube (CNT)/polydimethylsiloxane (PDMS) composite structures (CPCSs). (**a**) Mixing sugar, water, and CNTs. (**b**) Mixture casting into cases of desired shapes. (**c**) Separation of the sugar cube mixed with the CNTs from the mold and PDMS precursor infiltration in the cube sugar in a vacuum chamber. (**d**) PDMS thermal curing and dissolution of sugar with water.

We used the sugar templating process to fabricate a compressible, three-dimensional, interconnected, and conductive CPCS. Figure 1a–d shows the fabrication process of the CPCS. To fabricate the CPCS, sugar (CJ Cheiljedang, Korea), deionized (DI) water, and CNTs (CM-250, Hanhwa Nanotech Corp., Korea) were mixed in a beaker at a weight ratio of 30:1:0.01–0.3 (Figure 1a). The sugar, homogenously mixed with CNTs, was placed in cases of desired shapes (Figure 1b). By evaporating the water for 4 h at room temperature, some parts of the sugar grains stuck together and connected to form a sugar cube. The sugar cube was separated from the mold and placed onto a Petri dish. A PDMS precursor (Sylgard 184, Dow Corning Corp., USA), in which the resin and hardener were mixed in a weight ratio of 10:1, was poured into the Petri dish (Figure 1c). The empty spaces in the sugar cube were filled with the PDMS precursor due to the capillary force. The sugar cube filled with the PDMS precursor was placed in a vacuum oven and cured for 5 h at 70 °C. After the curing process, the sugar was dissolved in water (Figure 1d) and then dried in air. 

### 2.2. Characterization of Porous CNT/PDMS Composite Structure

Field emission scanning electron microscopy (FE-SEM, SUPRA 40VP, Carl Zeiss, Germany) was conducted to identify the structure of the fabricated CPCSs. The mechanical properties of the CPCSs were measured using a universal testing machine (JSV-H1000, JISC, Japan) with a load cell (HF-100, JISC, Tokyo, Japan). The sample was compressed at a speed of 10% strain/min, except for the strain rate test. To use the CPCS as pressure sensors, polyethylene terephthalate (PET) coated with indium-tin oxide films (Fine Chemical Industry, Korea) for electrodes were attached to the top and bottom of the CPCS and were electrically connected to the outside through wiring. To reduce the contact resistance between the fabricated CPCS and the electrodes, silver paste (P-100, Jin Chem, Korea) was used on both the top and bottom surfaces of the CPCSs. To evaluate the performance of the fabricated pressure sensors based on CPCS, the changes in the resistance between the top and bottom electrodes were measured with an LCR-meter (HIOKI-3536, HIOKI, Japan). The sensitivity, repeatability, hysteresis, and reliability of the CPCS pressure sensor were measured in real time by connecting the LCR-meter to the computer.

### 2.3. Working Principle

**Figure 2:**
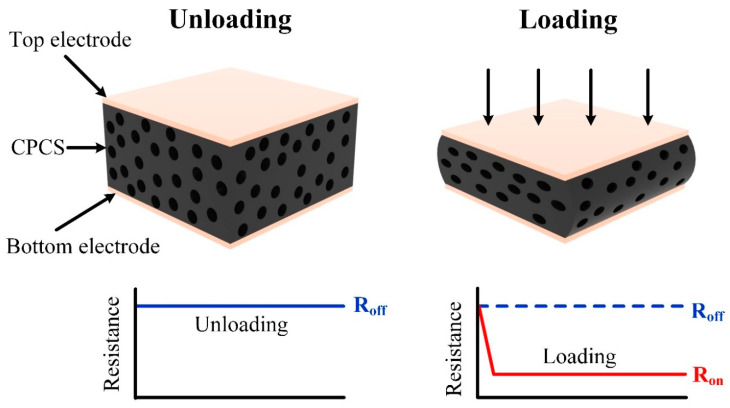
Working principle of the proposed pressure sensor. The resistance between the two electrodes changes with the applied pressure because of the increased conductive pathways.

Figure 2 shows the working mechanism of the pressure sensors based on the CPCS. The CPCS remains an initial resistance under the unloading condition. When a load is applied to the sensors, the pores inside the CPCS are compressed. As the pores are compressed, the randomly arrayed CNTs in the internal pores move closer or come into contact with each other. These increased conductive pathways decrease the resistance (negative piezoresistive effect) between the two electrodes, which can be measured. When the pressure is released, the CPCS fully recovers to its initial status due to its excellent mechanical properties, and the resistance changes back to the initial value.

## 3. Results

### 3.1. Surface Structures of the Fabricated CPCSs

**Figure 3:**
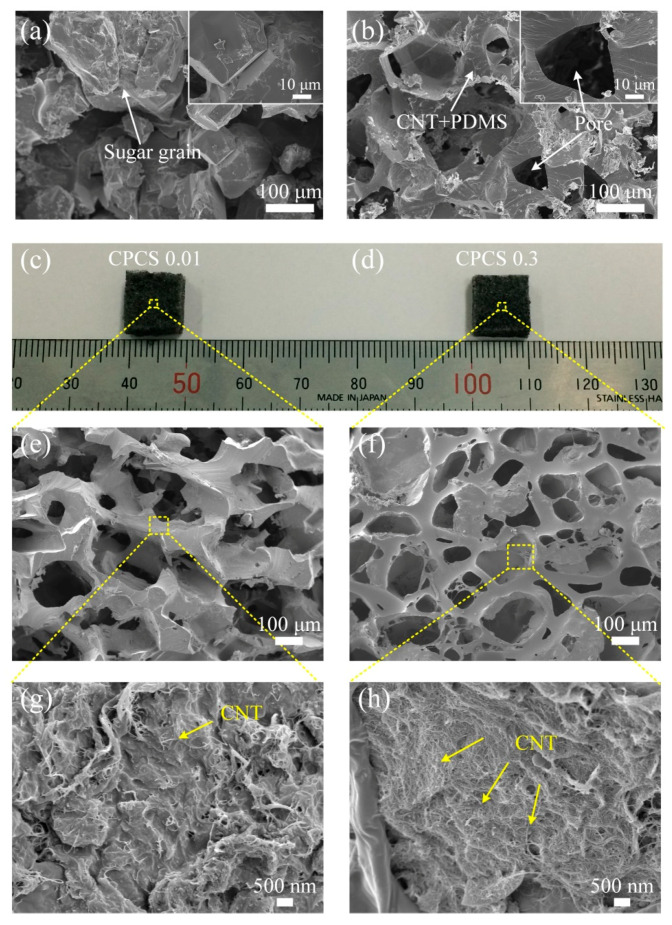
Photos and SEM images of the fabricated sugar cube and CPCS. (**a**) SEM image of the sugar cube. (**b**) SEM image of CPCSs. (**c**) Photograph of CPCS 0.01. (**d**) Photograph of CPCS 0.3. (**e**,**g**) SEM images of CPCS 0.01. (**f**,**h**) SEM images of CPCS 0.3. A relatively larger concentration of CNTs exists in CPCS 0.3 than that in CPCS 0.01.

As mentioned earlier, the highly compressible, three-dimensional, interconnected porous structure can be fabricated by using a simple method. The sugar cube is formed owing to sticking together of some sugar grains, followed by water evaporation (Figure 3a). After filling the empty space in the sugar cube with the PDMS precursor, the PDMS precursor was cured in an oven. The sugar was dissolved in water, and the pores of the CPCS became interconnected (Figure 3b). As this porous structure is easily compressed with applied pressure, it can be applied to various electronic and wearable systems because of its tunable mechanical and electrical properties. The porosity, the ratio of the empty space to total volume, depends on the empty spaces in the sugar cube. We measured the porosity of CPCSs to confirm the repeatability. The measured porosity was the average obtained from ten measurement results from each CPCS. The porosity of the CPCS was measured as 66.27% ± 0.56%. To demonstrate the ability to quantitatively control the concentration of CNTs, the CPCSs with different CNT concentration ratios (0.01, 0.3) were prepared (Figure 3c,d). CPCSs 0.01 and 0.3 are porous structures with CNT concentrations of 0.01 and 0.3, respectively. Figure 3e,f show scanning electron microscopy (SEM) images of the CPCS 0.01 and 0.3 surfaces. We verified that the concentration of CNTs on the CPCS increased with increasing CNT concentration during the fabrication process (Figure 3g,h). This indicates that the conductivity and mechanical properties of the CPCSs can be tuned by varying the concentration of CNTs. This method is also very simple and applicable over a large area compared to other methods such as drop casting [26,28], dip coating [29,30], and injection [31]. In cases of low CNT concentrations, CPCSs can be utilized as flexible sensors because of their high compressibility properties and low elastic modulus. Alternatively, in cases of high CNT concentrations, we can obtain a CPCS with low resistance (approximately 10 Ω) and highly stable mechanical and electrical properties. Because CNTs exhibit high tensile strength, an increased concentration of CNTs leads to the reinforcement of the mechanical strength of the CPCSs. Therefore, depending on the concentration of CNTs, CPCSs can be applied to wearable sensors and flexible conductors to impart different mechanical and electrical properties. 

### 3.2. Mechanical and Electrical Properties

**Figure 4:**
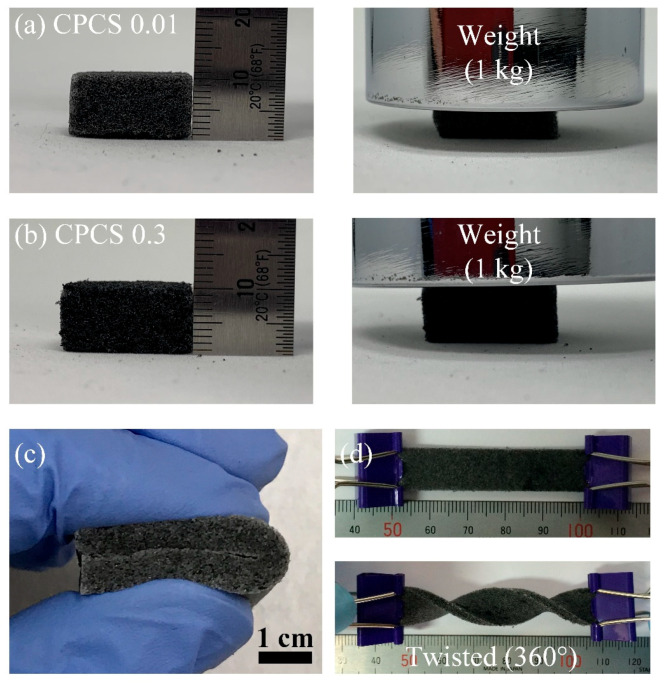
Photos captured before and after loading of a weight of 1 kg on the hexagonal-shaped (**a**) CPCS 0.01, and (**b**) CPCS 0.3. (**c**) Fully folded band-like CPCSs. (**d**) Non-twisted and twisted (360°) CPCSs.

Figure 4 shows the mechanical stability of CPCS when it was deformed into various shapes. As shown in Figure 4a, CPCS 0.01 was compressed more when a weight of 1 kg was placed on it, and the measured compressive strain was approximately 65%. On the contrary, the compressive strain of CPCS 0.3 was only approximately 25% when the same weight was applied on it (Figure 4b). As shown in Figure 4c,d, the CPCS returned to its original state without any damage, even when it was completely folded or twisted to 360°.

**Figure 5:**
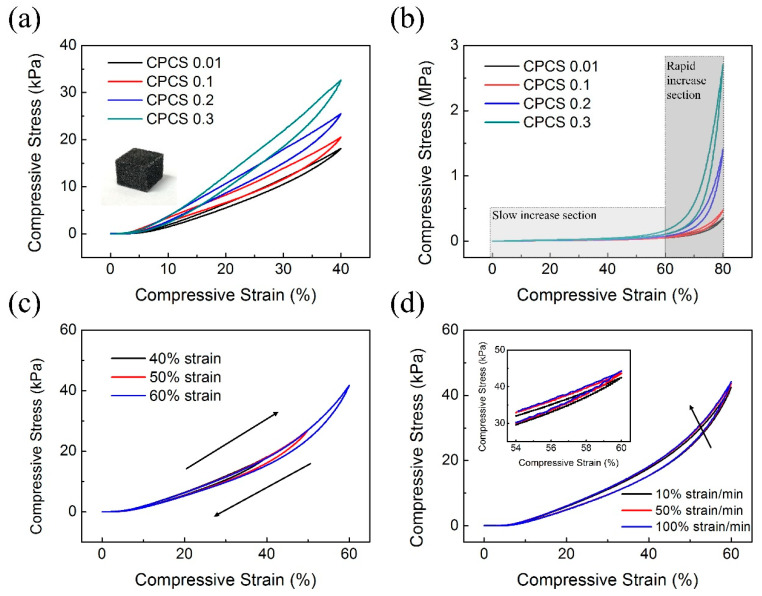
Mechanical characterization of the CPCS. Compressive stress–strain curves of CPCS with different concentrations of CNTs at (**a**) 40% (**b**) and 80% strain. After the pores of the CPCS were closed, the compressive stress rapidly increased. (**c**) Compressive stress–strain curves of CPCS 0.01 at different maximum strains of 40%, 50%, and 60%. (**d**) Curves tested under different strain rates Inset: enlarged graph from the 54% to 60% strain range.

To evaluate the mechanical properties of the CPCSs, compressive tests were quantitatively conducted under various conditions (i.e., CNT concentration, strain range, strain rate). Figure 5a,b show the measured compressive stress–strain curves for different CNT concentrations at 40% and 80% strain, respectively. The inset image shows the hexagonal-shaped CPCS for the evaluation of mechanical strength. As the CNT concentration increases, a higher pressure is necessary for compression. In the case of CPCS 0.01, at 80% strain, the maximum compressive deformation occurred at 0.35 MPa. However, in the case of CPCS 0.3, the maximum compressive stress increased, and the maximum compressive deformation occurred at approximately 2.7 MPa. These results imply that although a low concentration of CNTs does not significantly change the mechanical properties, a high concentration of CNTs dramatically enhances the elastic modulus of the CPCSs. Furthermore, the compressive stress–strain curves are largely divided into two sections: slow increase and rapid increase (Figure 5b). In the slow-increase section, four samples (CPCS 0.01, 0.1, 0.2, and 0.3) showed few differences in compressive stress up to 60% of compressive strain. The slow-increase section is where the CPCS is easily compressed even at low pressures for the pores existing inside the structure. Because the pores are easily deformed by pressure, high pressure is not required to compress the composite structure in the slow increase section. On the other hand, in the rapid increase section, the CPCS needs high pressure to compress the structure. After the pores are closed, the structure is changed to a solid composite structure. Therefore, much higher pressure is necessary to compress the CPCS in the rapid increase section, compared to that in the slow increase section. We also measured the compressive stress of CPCS 0.01 as a function of strain for the loading–unloading at a series of different strains (Figure 5c). The curves returned to the original points without signal degradation. Additionally, as the strain rate increased from 10% to 100%, the maximum compressive stress increased by only 3% (Figure 5d). These results mean that the CPCS completely recovers to its shape without any plastic deformation, and that the CPCS has high mechanical stability.

**Figure 6:**
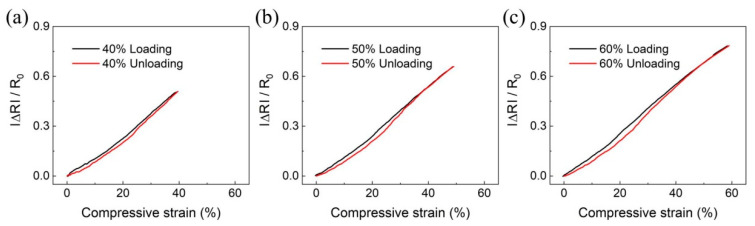
The relative resistance changes according to compressive strain with (**a**) 40%, (**b**) 50%, and (**c**) 60%. Black lines show changes in resistance when loading, and red lines show changes in resistance when unloading.

Hysteresis, which is the difference in the signal under loading and unloading of applied pressure, is still problematic in the piezoresistive pressure sensor. The sensor hysteresis is a critical factor in order to utilize the manufactured sensor in practical applications. To verify the hysteresis of the sensor, we measured the relative resistance change according to compressive strain using CPCS 0.01. The loading and unloading curves almost overlapped throughout the strain range (0~40%, 50%, 60%) (Figure 6). The maximum hysteresis error was about 9.47% at the 60% loading and unloading curves. These results indicate that the hysteresis error of the fabricated sensor is stable within 10%. 

**Figure 7:**
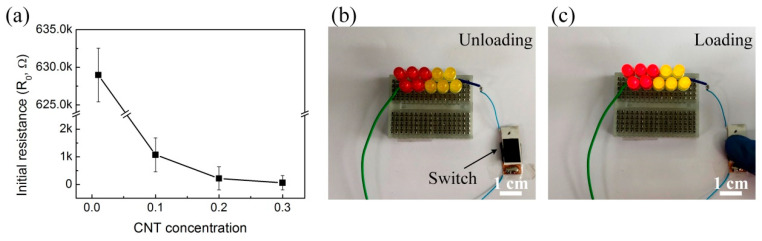
Electrical properties of the CPCS. (**a**) Change in initial resistance of CPCS according to the CNT concentration (from 0.01 to 0.3). (**b**) The CPCS used as a resistor switch. LED bulbs turned off when the CPCS switch was unloaded. (**c**) LED bulbs turned on as soon as the CPCS switch was pressed.

As shown in Figure 7a, we measured the resistance of CPCS according to the CNT concentration. As the CNT concentration increased, the resistance decreased sharply. In particular, CPCS 0.1 showed a sharp decrease in resistance compared to CPCS 0.01. For CPCS samples with a CNT concentration above 0.1, the initial resistance was rapidly stabilized and there was no significant change according to the CNT concentration. The trend of a sharp decrease as a function of the CNT concentration agrees well with the percolation theory, which exponentially increases the electrical conductivity [3,23,32,33]. This result indicates that CPCS 0.01, which has the highest initial resistance, is expected to have the largest resistance change under pressure. We applied CPCS 0.01 as an elastomeric switch, as shown in Figure 7b,c. A CPCS of 0.01 was connected to a light-emitting diode (LED) and a power supply in series. When 5 V was applied, the brightness of the light was kept low in the unloading (initial) state for the high resistance (Figure 7b). However, the LEDs became brighter as the loading force was applied with decreased resistance (Figure 7c). This shows that CPCS resistance significantly varies with the applied pressure. 

### 3.3. Sensor Evaluation

**Figure 8:**
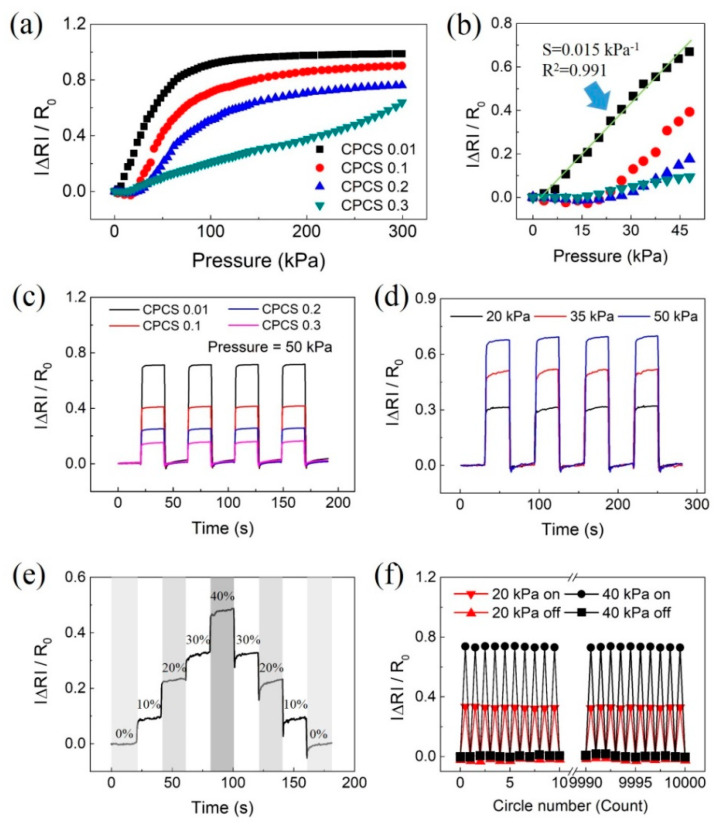
Performance evaluation of flexible pressure sensors based on CPCS. (**a**,**b**) The measured relative resistance changes as a function of the applied pressure for sensors with different CNT concentrations. The sensor based on CPCS 0.01 showed the highest sensitivity of 0.015 kPa^−1^ and a linearity of 0.991. (**c**) Measurement results under repeated loading–unloading tests with different CNT concentrations at 50 kPa pressure. (**d**) With different pressures at the sensor based on CPCS 0.01. (**e**) Dynamic responses of the pressure sensor at continuously increasing and decreasing pressures. (**f**) Reliability test results: black squares (■) and red triangles (▲) show changes in resistance when released, and black circles (●) and red inverted triangles (■) show changes in the resistance when pressed.

We conducted an evaluation to confirm the performance of pressure sensors based on the CPCS. Figure 8a is a graph that shows changes in the relative resistance change rate according to the applied pressure. The sensitivity S of the sensor is defined as *S = (*Δ*R/R_i_)/*Δ*P*, where *p* represents the applied pressure, and *R_a_* and *R_i_* indicate the resistances with and without applied pressure, respectively. The sensor based on CPCS 0.01 showed the highest sensitivity of 0.015 kPa^−1^ under 50 kPa. As the CNT concentration was increased, the sensitivity of the sensor decreased, and CPCS 0.01 showed a 10 times higher sensitivity than CPCS 0.3 at 50 kPa. As mentioned before, the sensitivity is related to the micropores of the structure. CPCS 0.01, which has low mechanical properties, is easily compressed by pressure, resulting in a rapid increase in contact between the CNTs. On the other hand, CPCS 0.3 has a relatively high elastic modulus because of the reinforced mechanical strength. Therefore, as the CNT concentration increased, the sensitivity of the sensor decreased rapidly. The sensor based on CPCS 0.01 also showed a high linearity of 0.991 from 0 to 50 kPa (Figure 8b). CPCS 0.01 showed the largest resistance change even when a repeated pressure of 50 kPa was applied to the sensors with different CNT concentrations (Figure 8c). We conducted an additional evaluation using CPCS 0.01 with the highest sensitivity and linearity. To compare the response capability, pressure sensors based on CPCS were subjected to pressures of 20, 35, and 50 kPa, as shown in Figure 8d. The sensor showed stable resistance responses under repeated pressure, and it returned to its initial state after removing the pressure. The pressure sensor based on CPCS showed high sensitivity and repeatability. We measured the relative resistance changes to evaluate the hysteresis and stability. As shown in Figure 8e, the resistance change values remained stable without any signal drift. The pressure sensor showed little hysteresis at each strain rate because of the viscoelastic properties of the PDMS. Figure 8f shows the results of the reliability evaluation of the sensors using CPCS 0.01. Pressures of 20 and 40 kPa were applied 10,000 times repeatedly to the fabricated sensors. The resistance of the first and last 10 cycles was measured when the pressure was applied and removed. The results show a stable response upon repeated loading and unloading cycles without any significant drift, verifying the excellent stability and reliability of the sensor.

**Figure 9:**
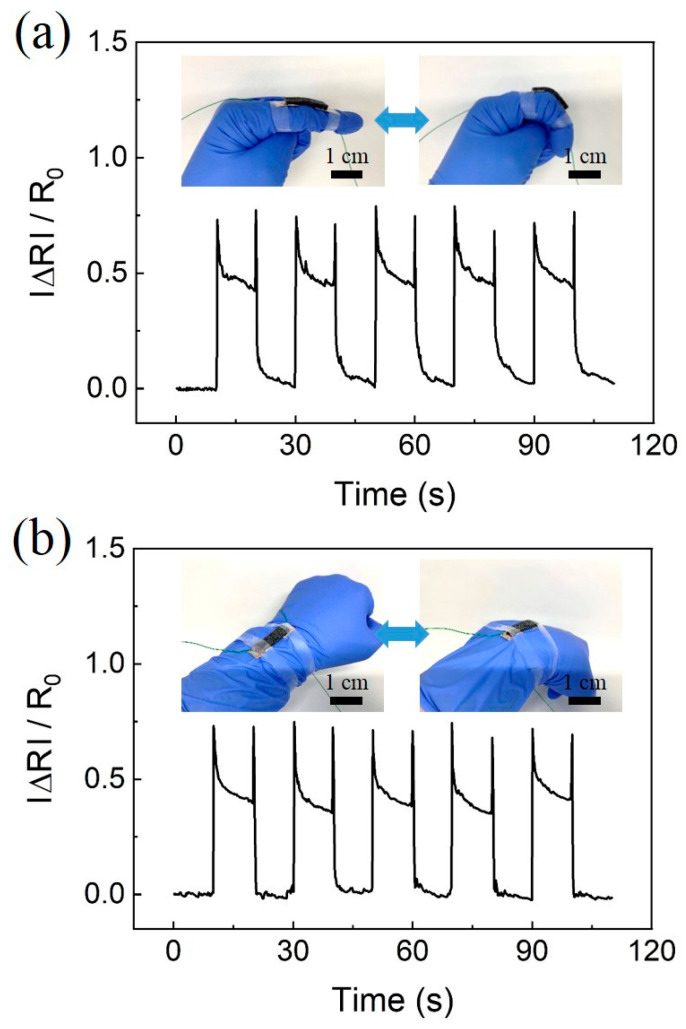
Demonstration of the applicability of pressure sensors based on CPCS. (**a**) Measurement of human motion during finger bending. (**b**) Measurement of human motion during wrist bending. The insets show the images of the sensors attached to the finger and wrist. The sensor is in relaxed and bent states, which correspond to bending angles of 0° and 30°, respectively.

As shown in Figure 9a,b, sensors were attached to the finger and wrist. The changes in relative resistance due to bending motions were monitored simultaneously. We repeatedly measured the resistance variation when the finger and wrist were bent for 10 s (Figure 9a,b). The inset images show the sensors attached to the finger and wrist in the relaxed and bent states, which correspond to bending angles of 0° and 30°. During bending, when the CPSC-based sensor was stretched, the resistance value sharply increased to a certain point and then partially recovered. On the contrary, during release, when the stretched CPCS was released and returned to its original state, the initial resistance value was recovered. The instantaneous peak signal that occurs during bending and releasing is caused by a larger deformation when it has a certain speed. After the motion of the finger (wrist) stopped, an equilibrium state stabilized the signal [7].

## 4. Conclusions

We fabricated facile piezoresistive pressure sensors based on the CNTs/PDMS composite structure via a low-cost and simple process. Through a simple sugar templating process, porous CPCSs with strong mechanical properties and conductivity were fabricated. We demonstrated that the mechanical and electrical properties could be controlled by tuning the concentration of CNTs. The flexible pressure sensor showed a high sensitivity of 0.015 kPa^−1^ and linearity of 0.991 at 50 kPa. Moreover, the as-prepared sensor exhibited low hysteresis, excellent repeatability, and high reliability. Furthermore, the pressure sensor could also distinguish bending motions, such as those of fingers and wrists. We believe that the CPCS-based pressure sensor, with high linear sensitivity, excellent stability, and good dynamic sensing capability, is a promising candidate for wearable devices and systems.

## References

[1] Wang X., Dong L., Zhang H., Yu R., Pan C., Wang Z.L. (2015). Recent Progress in Electronic Skin. Adv. Sci..

[2] Chortos A., Liu J., Bao Z. (2016). Pursuing prosthetic electronic skin. Nat. Mater..

[3] Tee B.C.-K., Wang C., Allen R., Bao Z. (2012). An electrically and mechanically self-healing composite with pressure- and flexion-sensitive properties for electronic skin applications. Nat. Nanotechnol..

[4] Jung S., Kim J.H., Kim J., Choi S., Lee J., Park I., Hyeon T., Kim D.H. (2014). Reverse-micelle-induced porous pressure-sensitive rubber for wearable human-machine interfaces. Adv. Mater..

[5] Liu C., Choi J. An Embedded PDMS Nanocomposite Strain Sensor toward Biomedical Applications. Proceedings of the 31st Annual International Conference of the IEEE Engineering in Medicine and Biology Society.

[6] Kim K., Choi J., Jeong Y., Cho I., Kim M., Kim S., Oh Y., Park I. (2019). Highly Sensitive and Wearable Liquid Metal-Based Pressure Sensor for Health Monitoring Applications: Integration of a 3D-Printed Microbump Array with the Microchannel. Adv. Healthc. Mater..

[7] Zhang M., Wang C., Wang H., Jian M., Hao X., Zhang Y. (2017). Carbonized Cotton Fabric for High-Performance Wearable Strain Sensors. Adv. Funct. Mater..

[8] Gong T., Zhang H., Huang W., Mao L., Ke Y., Gao M., Yu B. (2018). Highly responsive flexible strain sensor using polystyrene nanoparticle doped reduced graphene oxide for human health monitoring. Carbon.

[9] Zhong W., Ding X., Li W., Shen C., Yadav A., Chen Y., Bao M., Jiang H., Wang D. (2019). Facile fabrication of conductive graphene/polyurethane foam composite and its application on flexible piezo-resistive sensors. Polymers.

[10] Xu M., Gao Y., Yu G., Lu C., Tan J., Xuan F. (2018). Flexible pressure sensor using carbon nanotube-wrapped polydimethylsiloxane microspheres for tactile sensing. Sens. Actuators A Phys..

[11] Pan L., Chortos A., Yu G., Wang Y., Isaacson S., Allen R., Shi Y., Dauskardt R., Bao Z. (2014). An ultra-sensitive resistive pressure sensor based on hollow-sphere microstructure induced elasticity in conducting polymer film. Nat. Commun..

[12] Li X., Huang W., Yao G., Gao M., Wei X., Liu Z., Zhang H., Gong T., Yu B. (2017). Highly sensitive flexible tactile sensors based on microstructured multiwall carbon nanotube arrays. Scr. Mater..

[13] Zhu B., Niu Z., Wang H., Leow W.R., Wang H., Li Y., Zheng L., Wei J., Huo F., Chen X. (2014). Microstructured graphene arrays for highly sensitive flexible tactile sensors. Small.

[14] Atalay O., Atalay A., Gafford J., Walsh C. (2018). A Highly Sensitive Capacitive-Based Soft Pressure Sensor Based on a Conductive Fabric and a Microporous Dielectric Layer. Adv. Mater. Technol..

[15] Guo Z., Mo L., Ding Y., Zhang Q., Meng X., Wu Z., Chen Y., Cao M., Wang W., Li L. (2019). Printed and flexible capacitive pressure sensor with carbon nanotubes based composite dielectric layer. Micromachines.

[16] Yoon J.I., Choi K.S., Chang S.P. (2017). A novel means of fabricating microporous structures for the dielectric layers of capacitive pressure sensor. Microelectron. Eng..

[17] Mannsfeld S.C.B., Tee B.C.K., Stoltenberg R.M., Chen C.V.H.H., Barman S., Muir B.V.O., Sokolov A.N., Reese C., Bao Z. (2010). Highly sensitive flexible pressure sensors with microstructured rubber dielectric layers. Nat. Mater..

[18] Chen Z., Wang Z., Li X., Lin Y., Luo N., Long M., Zhao N., Xu J. (2017). Bin Flexible Piezoelectric-Induced Pressure Sensors for Static Measurements Based on Nanowires/Graphene Heterostructures. ACS Nano.

[19] Gao Q., Meguro H., Okamoto S., Kimura M. (2012). Flexible Tactile Sensor Using the Reversible Deformation of Poly(3-hexylthiophene) Nanofiber Assemblies. Langmuir.

[20] Lee K.Y., Yoon H.J., Jiang T., Wen X., Seung W., Kim S.W., Wang Z.L. (2016). Fully Packaged Self-Powered Triboelectric Pressure Sensor Using Hemispheres-Array. Adv. Energy Mater..

[21] Meng B., Tang W., Too Z.H., Zhang X., Han M., Liu W., Zhang H. (2013). A transparent single-friction-surface triboelectric generator and self-powered touch sensor. Energy Environ. Sci..

[22] Janczak D., Słoma M., Wróblewski G., Młożniak A., Jakubowska M. (2014). Screen-printed resistive pressure sensors containing graphene nanoplatelets and carbon nanotubes. Sensors.

[23] Zhao H., Bai J. (2015). Highly sensitive piezo-resistive graphite nanoplatelet-carbon nanotube hybrids/polydimethylsilicone composites with improved conductive network construction. ACS Appl. Mater. Interfaces.

[24] Jung Y., Jung K., Park B., Choi J., Kim D., Park J., Ko J., Cho H. (2019). Wearable piezoresistive strain sensor based on graphene-coated three-dimensional micro-porous PDMS sponge. Micro Nano Syst. Lett..

[25] Zhang X.C., Scarpa F., McHale R., Limmack A.P., Peng H.X. (2016). Carbon nano-ink coated open cell polyurethane foam with micro-architectured multilayer skeleton for damping applications. RSC Adv..

[26] Han J., Kim B., Li J., Meyyappan M. (2013). Flexible, compressible, hydrophobic, floatable, and conductive carbon nanotube-polymer sponge. Appl. Phys. Lett..

[27] Yao H., Ge J., Wang C., Wang X. (2013). A flexible and highly pressure-sensitive graphene-polyurethane sponge based on fractured microstructure design. Adv. Mater..

[28] Song Y., Chen H., Su Z., Chen X., Miao L., Zhang J., Cheng X., Zhang H. (2017). Highly Compressible Integrated Supercapacitor–Piezoresistance-Sensor System with CNT–PDMS Sponge for Health Monitoring. Small.

[29] Nguyen D.D., Tai N.-H., Lee S.-B., Kuo W.-S. (2012). Superhydrophobic and superoleophilic properties of graphene-based sponges fabricated using a facile dip coating method. Energy Environ. Sci..

[30] Wang C.F., Lin S.J. (2013). Robust Superhydrophobic/Superoleophilic Sponge for Effective Continuous Absorption and Expulsion of Oil Pollutants from Water. ACS Appl. Mater. Interfaces.

[31] Tran D.N.H., Kabiri S., Sim T.R., Losic D. (2015). Selective adsorption of oil–water mixtures using polydimethylsiloxane (PDMS)–graphene sponges. Environ. Sci. Water Res. Technol..

[32] Chang L., Friedrich K., Ye L., Toro P. (2009). Evaluation and visualization of the percolating networks in multi-wall carbon nanotube/epoxy composites. J. Mater. Sci..

[33] Rul S., Lefèvre-Schlick F., Capria E., Laurent C., Peigney A. (2004). Percolation of single-walled carbon nanotubes in ceramic matrix nanocomposites. Acta Mater..

